# A marine analgesic peptide, Contulakin-G, and neurotensin are distinct agonists for neurotensin receptors: uncovering structural determinants of desensitization properties

**DOI:** 10.3389/fphar.2015.00011

**Published:** 2015-02-10

**Authors:** Hee-Kyoung Lee, Liuyin Zhang, Misty D. Smith, Aleksandra Walewska, Nadeem A. Vellore, Riccardo Baron, J. Michael McIntosh, H. Steve White, Baldomero M. Olivera, Grzegorz Bulaj

**Affiliations:** ^1^Department of Medicinal Chemistry, College of Pharmacy, Skaggs Research Institute, University of UtahSalt Lake City, UT, USA; ^2^Department of Pharmacology and Toxicology, University of UtahSalt Lake City, UT, USA; ^3^Faculty of Chemistry, University of GdanskGdansk, Poland; ^4^Department of Biology, University of UtahSalt Lake City, UT, USA; ^5^Department of Psychiatry, University of UtahSalt Lake City, UT, USA

**Keywords:** *Conus peptides*, conotoxin, neuropeptides, neurotensin, neurotensin receptors, GPCRs, receptor internalization, pain

## Abstract

Neurotensin receptors have been studied as molecular targets for the treatment of pain, schizophrenia, addiction, or cancer. Neurotensin (NT) and Contulakin-G, a glycopeptide isolated from a predatory cone snail *Conus geographus*, share a sequence similarity at the C-terminus, which is critical for activation of neurotensin receptors. Both peptides are potent analgesics, although affinity and agonist potency of Contulakin-G toward neurotensin receptors are significantly lower, as compared to those for NT. In this work, we show that the weaker agonist properties of Contulakin-G result in inducing significantly less desensitization of neurotensin receptors and preserving their cell-surface density. Structure-activity relationship (SAR) studies suggested that both glycosylation and charged amino acid residues in Contulakin-G or NT played important roles in desensitizing neurotensin receptors. Computational modeling studies of human neurotensin receptor NTS1 and Contulakin-G confirmed the role of glycosylation in weakening interactions with the receptors. Based on available SAR data, we designed, synthesized, and characterized an analog of Contulakin-G in which the glycosylated amino acid residue, Gal-GalNAc-Thr10, was replaced by memantine-Glu10 residue. This analog exhibited comparable agonist potency and weaker desensitization properties as compared to that of Contulakin-G, while producing analgesia in the animal model of acute pain following systemic administration. We discuss our study in the context of feasibility and safety of developing NT therapeutic agents with improved penetration across the blood-brain barrier. Our work supports engineering peptide-based agonists with diverse abilities to desensitize G-protein coupled receptors and further emphasizes opportunities for conotoxins as novel pharmacological tools and drug candidates.

## INTRODUCTION

**FIGURE 1:**
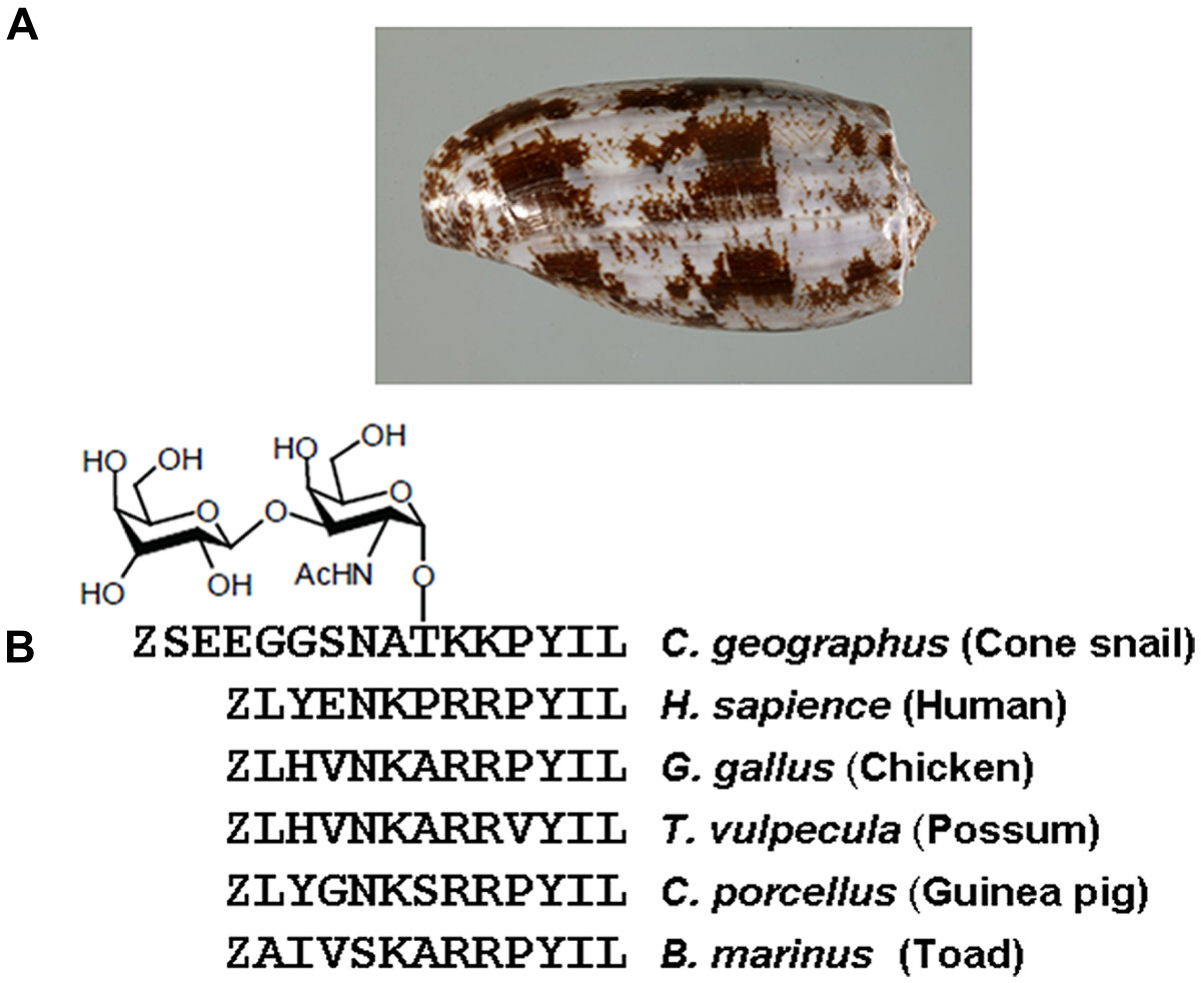
**(A)** A shell of predatory marine snail *Conus geographus* from which Contulakin-G was originally isolated. **(B)** Comparison of Contulakin-G with neurotensin derived from various vertebrate animals. The peptides share a similarity in the C-terminal part of the sequence which is critical for interactions with neurotensin receptors.

Contulakin-G was discovered over 15 years ago as a member of the neurotensin (NT) family from the venom of predatory marine snail, *Conus geographus* (**Figure 1A**; Craig et al., 1999). Contulakin-G is a 16 amino acid peptide with two post-translational modifications: pyroglutamate (Z) at the N-terminus, and β-D-Gal-(1→3)-α-D-GalNAc-(1→) disaccharide attached to Thr10 (**Figure 1B**). Contulakin-G exhibited potent analgesic activity in three pain models in rats following intrathecal delivery, namely in tail-flick (acute pain), formalin test, and CFA-induced allodynia inflammatory pain (Craig et al., 2002; Han et al., 2008). Both NT and Contulakin-G exhibited comparable potencies in a rat formalin assay (ED_50_ for Contulakin-G was 0.07 nmol (Allen et al., 2007), while ED_50_ for NT was 0.11 nmol (Roussy et al., 2008). In mice, the analgesic potency of Contulakin-G (ED_50_ = 1 pmol) was 600 times higher than that of NT in the formalin assay following intrathecal administration (Craig et al., 2002; Han et al., 2008). Contulakin-G (coded as CGX-1160) was granted an orphan drug designation by the US Food and Drug Administration (FDA) and reached a clinical development stage for the treatment of chronic intractable pain following intrathecal administration in patients with spinal cord injury (Business Wire, August 30th 2005).

The C-terminal sequence of Contulakin-G shares a similarity with an endogenous NT found in vertebrate animals (**Figure 1B**). NT is a 13 amino acid neuropeptide involved in a variety of central and peripheral neuromodulatory effects (Nemeroff et al., 1992; Vincent et al., 1999; Dobner, 2005; Boules et al., 2006). Pleiotropic properties of NT are supported by its involvement in Parkinson’s disease, nociception, cancer, blood pressure, glucose control, autism spectrum disorders, appetite, and feeding (Mazella et al., 2012; Boules et al., 2013, 2014; Kleczkowska and Lipkowski, 2013). NT also plays a role in the pathophysiology of mental diseases (Boules et al., 2013, 2014). Metabolically stable NT analogs that penetrate the blood-brain-barrier (BBB) could be used for the treatment of pain, schizophrenia, or substance abuse (Boules et al., 2006; Dobner, 2006). Several NT analogs exhibit potent antinociceptive activities (al-Rodhan et al., 1991; Gui et al., 2004; Dobner, 2006), whereas our group showed that glycosylated or lipidated NT analogs also exhibit potent anticonvulsant activities (Lee et al., 2009; Green et al., 2010). Branched NT analogs have anticancer (Falciani et al., 2010, 2013a,b) and anti-apoptotic activities (Devader et al., 2013). Recent advances in developing new agonists for NT receptors include NTS-1 selective small-molecules (Peddibhotla et al., 2013; Di Fruscia et al., 2014; Hershberger et al., 2014) and NTS2-selective mimetics (Einsiedel et al., 2011; Held et al., 2013). Pleiotropic nature of NT includes promoting progression of certain types of cancer (Wu et al., 2013), providing new challenges and opportunities for preclinical and clinical development of NT-based analogs. Taken together, NT analogs are pharmacological tools and potential therapeutic agents for a variety of medical conditions which involve neurotensin receptors.

Contulakin-G was previously shown to be an agonist for all three subtypes of neurotensin receptors, NTS1, NTS2, and NTS3 with submicromolar potency (Craig et al., 1999). By measuring phosphoinositide accumulation in CHO cells expressing hNTS1, Craig et al. (1999) determined the agonist potency of Contulakin-G and NT as 0.96 μM and 1.4 nM, respectively. We hypothesized that the weaker-agonist property of Contulakin-G can result in decreased neurotensin receptor desensitization, hence improving its analgesic properties due to preserving the target receptor occupancy. Desensitization of neurotensin receptors was extensively studied in various cells (Souaze et al., 1997, 2006; Vandenbulcke et al., 2000; Souaze, 2001; Mazella and Vincent, 2006; Souaze and Forgez, 2006), while the weaker-agonist phenomenon was previously described for various GPCRs (Clark et al., 1999). To test this hypothesis, we studied structure-agonist relationships for Contulakin-G and NT using endogenously expressed NT receptors in human colonic adenocarcinoma HT-29 cells (Amar et al., 1986; Turner et al., 1990). Here we report that: (1) Contulakin-G is a weaker agonist exhibiting significantly lower desensitization potency, as compared to that of NT, and (2) both glycosylation and charged amino acid residues contribute to desensitization properties of Contulakin-G and NT, and (3) SAR results support engineering neuropeptide-based agonists with diverse agonist and desensitization potencies. Our work provides a basis for engineering novel pharmacological tools for neurotensin receptors with varying desensitization properties.

## MATERIALS AND METHODS

### GENERAL SYNTHETIC PROCEDURES

Fmoc-amino acids were purchased from Chem–impex International Inc. Reagents, chemicals, and memantine HCl, were obtained from Aldrich Chemical Corporation and used without further purification. Fmoc-Leu-Wang resin (0.57 meq/g) was obtained from Peptide International Inc. Fmoc-Thr(α-TF-Ac_6_)-OH was obtained from Sussex Research Laboratories Inc. Peptides were synthesized automatically on Symphony Peptide Synthesizer (Protein technology Inc) or Apex 396 Peptide Synthesizer (AAPPTec Inc). Fmoc-protected amino acids (fivefold) were coupled automatically onto Fmoc-Leu-Wang resin by PyBop method (Fmoc-amino acid/PyBop/DIPEA, 1:0.98:2, molar ratio). Manual coupling reactions were performed under N_2_ atmosphere, unless otherwise indicated. Peptide purification was carried out using a semi-preparative diphenyl column (Vydac, 219TP101522) or a semi-preparative C18 column (Vydac, 218TP510) on a Waters 600 pump system equipped with a Waters 2487 dual wavelength detector (*λ*_1_ = 220 nm, *λ*_2_ = 280 nm). The purities of peptides were determined on a Vydac diphenyl column (218TP54) in Waters Alliance 2695 system unless indicated otherwise. The HPLC mobile phases were: buffer A, water (0.1% TFA), and buffer B, 90% acetonitrile in water (0.1% TFA). Peptides were quantified on a Cary 50 Bio UV-visible spectrophotometer. Peptide metabolic stability was monitored using YMC ODS-A S-5 120 Å column (AA12S052503WT) and Waters Alliance 2695 system. Metabolic stability assays were performed using an Eppendorf thermomixer. Peptide identities were verified by MALDI-TOF MS at the University of Utah Core Facility.

### CHEMICAL SYNTHESIS OF CONTULAKIN-G

Chemical syntheses of Contulakin-G and its glycosylated analogs were previously published (Craig et al., 1999; Westerlind and Norberg, 2006). Contulakin-G was synthesized on an Apex 396 automated peptide synthesizer (AAPPTec) on 30 μmol scale applying standard solid-phase Fmoc (9-fluorenylmethyloxycarbonyl) protocols. The peptide was constructed on a preloaded Fmoc-L-Leu-Wang resin. 10-fold excess of amino acids were used. Coupling activation was achieved with 1 equivalent of 0.4 M benzotriazol-1-yl-oxytripyrrolidinophosphonium hexafluorophosphate (PyBOP) and 2 equivalents of 2 M N,N-diisopropylethyl amine (DIPEA) in N-methyl-2-pyrrolidone (NMP) as the solvent. Each coupling reaction was conducted for 60 min. Fmoc deprotection was carried out for 20 min with 20% piperidine in dimethylformamide (DMF). Fmoc-Thr(*α*-TF-Ac6)-OH (1.25-fold) was manually coupled on the resin for 2 h. After assembling all the amino acids, Contulakin-G was removed from the resin by a 3.5 h treatment with 0.5 mL of Reagent K (TFA/water/phenol/thioanisole/1,2-ethanedithiol 82.5/5/5/5/2.5 by volume) and subsequently filtered and precipitated with 10 mL of cold methyl-tert-butyl ether (MTBE). The crude peptide was then collected by centrifugation at 7,000 ×*g* for 4 min and washed once with 10 mL of cold MTBE. The washed peptide pellet was dissolved in 10% acetonitrile in 0.1% TFA in water and purified by reversed-phase (RP) HPLC using a semi-preparative C18 Vydac column (218TP510, 250 mm × 10 mm, 5-μm particle size) eluted with a linear gradient ranging from 15 to 55% of solvent B, at a flow rate 4 ml/min. The eluent was monitored by measuring absorbance at 220 nm. Purity of the peptide was assessed by an analytical C18 Vydac reversed-phase HPLC (218TP54, 250 mm × 4.6 mm, 5 μm particle size) using a linear gradient ranging from 5 to 65% of solvent B in 30 min with a flow rate 1 ml/min. In the next step, deacetylation reaction of Thr(α-TF-Ac6) was performed with 50 mM of sodium methoxide in methanol for 2.5 h, at RT. The progress of deacetylation was monitored by RP-HPLC. The peptide was purified again using the same method as described above. Molecular mass of Contulakin-G was confirmed by MALDI-TOF mass spectrometry. Final yield was 3.5%.

### SYNTHESIS OF CONTULAKIN-G-MEMANTINE

Fmoc-protected amino acids (fivefold) were coupled automatically onto Fmoc-L-Leu-Wang resin (0.57 meq, 50 μmol scale) by PyBop method (Fmoc-amino acid/PyBop/DIPEA, 1:0.98:2, molar ratio) on Symphony Peptide Synthesizer. Fmoc-Glu(OAll)-OH was assembled at the10th position. After coupling all the amino acids, the resin was treated with HOAc (0.25 mL), *N*-methylmorpholine (0.125 mL), and CH_2_Cl_2_ (5 mL), and Pd^0^(PPh_3_)_4_ (0.32 g, 0.277 mmol) under Nitrogen protection for 2 h to remove the allyl protecting group of Glu^10^. The resin was washed with CH_2_Cl_2_ and neutralized with DIPEA. Excess Palladium residues were removed after treating the resin with 0.02 M sodium diethyldithiocarbamate in DMF solution. The resin was washed again with DMF and CH_2_Cl_2_. The acid group of Glu^10^ was activated with one-fold of PyBOP (26 mg, 50 μmol, HOBt (6.75 mg, 50 μmol) and DIPEA (26 μL, 150 μmol) for 5 min, followed by the addition of memantine hydrochloride (10.8 mg, 50 μmol), and shaken for 24 h. The peptide was cleaved from the resin with Reagent K and was precipitated out of MTBE. The crude peptide was purified with semi-preparative HPLC (Vydac diphenyl column, 219TP101522). The buffer A and the buffer B were used to produce a linear gradient from 5 to 50% of buffer B over 50 min with a flow rate of 10 mL/min. The elution was monitored by UV detection at 220 nm. Purified analogs were quantified by measuring UV absorbance at 274.6 nm (molar absorbance coefficient ε = 1420.2 cm^-1^M^-1^). Peptide purification was monitored using an Alliance HPLC system with a linear gradient from 5 to 95% buffer B over 30 min. The purity of the final product was >95%. A scheme for the chemical synthesis is provided in the Supplemental Material (Figure S1). Final yield was 20%.

### CALCIUM ACTIVATION ASSAY

HT-29 cells were seeded into black-sided, clear bottom, cell-culture treated 96-well plates for each assay. The cells were seeded at 45,000–50,000 cells per well and grown overnight until 95% confluent. The growth media consisted of DMEM supplemented with 10% FBS, 4 mM L-glutamine, and 20 mM HEPES. The cells were loaded with 1 μM of Fluo-4-NW (Life Technologies, Grand Island, NY, USA) and incubated in the dark at room temperature for an hour. Then, the cells were exposed to the Contulakin-G analogs (10 μM–10 pM), and the fluorescence was measured as Arbitrary Fluorescence Units (AFU) by the scanning microplate fluorometer Flexstation (Molecular Devices, Sunnyvale, CA, USA). Results were expressed as a percentage of the 1 μM peak response. Fluorescence data from quadruplicate experiments were analyzed using Graphpad Prism 3.0, and EC_50_ values were calculated.

### DESENSITIZATION OF NEUROTENSIN RECEPTOR ACTIVATION

Desensitization of the NTR-mediated functional response (calcium mobilization) following exposure to Contulakin-G and NT was evaluated in HT-29. The cells were exposed to Contulakin-G or NT for 10 min (10 μM–100 pM). After exposure to Contulakin-G or NT, cells were washed with PBS and incubated in growth media for 15 min in the absence of peptide. The cells were loaded with the calcium sensitive dye Fluo-4-NW (Life Technologies, Grand Island, NY, USA) and incubated in the dark at room temperature for an hour. During the calcium mobilization assay, the cells were re-exposed to the same peptide (at 1 μM only) and increased fluorescence was detected using the scanning microplate fluorometer Flexstation (Molecular Devices, Sunnyvale, CA, USA). DC_50_ values for desensitization calculated from quadruplicate experiments using Graphpad Prism 3.0. Results were expressed as a percentage of the 1 μM peak response in the control cells (not previously exposed to any peptide).

### DETERMINATION OF CELL SURFACE DENSITY OF NTR

To measure the recovery of cell-surface receptors upon the activation, binding assay was employed using Europium-labeled NT (Eu-NT) as a ligand (PerkinElmer, Waltham, MA, USA). The higher fluorescence signal would imply more NTR on the cell surface. The HT-29 cells were exposed to the agonists for 10 min. At the indicated time point, cells were washed with media and the binding assay was performed in quadruplicate. Eu-NT and ligands were diluted in binding buffer (50 mM Tris-HCl pH 7.5, 5 mM MgCl_2_, 25 mM EDTA, 0.2% BSA). Samples were incubated at room temperature for 90 min in a total volume of 200 μL. Following incubation, samples were washed four times with wash buffer (50 mM Tris-HCl pH 7.5, 5 mM MgCl_2_). Enhancement solution (200 μL) was added, and the plates were incubated at room temperature for 30 min. The plates were read on a Wallace VICTOR^3^ instrument using the standard Eu-TRF measurement (λ_ex_ = 340 nm, 400 μs decay, and λ_em_ = 615 nm for 400 μs). Competition curves were analyzed from quadruplicate experiments with GraphPad Prism using the sigmoidal concentration–response (variable slope) classical equation for non-linear regression analysis.

### TAIL FLICK TEST

Pain sensitivity in the tail flick assay was measured using a radiant heat beam focused on the animal’s tail while it was on an automated Plantar/Tail Analgesic Meter, Series 8 (Model 336TG; IITC, Woodland Hills, CA, USA). The latency, in seconds, to the tail flick response was recorded as a measure of the acute thermal pain threshold. The test substance was administered intraperitoneally (i.p.,), at a volume of 0.1 ml/10g body weight using a 1 mL syringe with a 26G 3/8 bevel needle. Immediately before the test, the mouse was habituated to the plexiglass restraining tube with its tail protruding for 2–3 min before the test. Tails were stimulated at ∼3 cm from tip. For the tail flick test, once the power source was manually triggered, a radiant heat beam of light was applied to the tail and the latency of the mouse to remove its tail from the heat source was automatically recorded at the moment the tail flick breaks the beam of light. The latency was analyzed for each mouse tested and average latency to tail withdrawal ±SEM. determined for each group. These values were compared statistically by Student’s *t*-test (two groups) or by one-way ANOVA (three or more groups) and considered significantly different if the *p*-value was less than 0.05. An animal receiving the requisite volume of vehicle was alternated with each mouse given the test drug (*n* = 8 per group). All animals were allowed free access to both food (Prolab RMH 3000) and water except when they were removed from their cages for the experimental procedure. All mice were housed, fed, and handled in a manner consistent with the recommendations in the National Research Council Publication, “Guide for the Care and Use of Laboratory Animals.”

### HOT PLATE TEST

Mice were brought to the testing room and allowed to acclimatize for 10 min before the test begins. Pain reflexes in response to a thermal stimulus were measured using a Hot Plate Analgesia Meter from IITC Instruments (IITC, Woodland Hills, CA, USA). The surface of the hot plate was heated to a constant temperature of 55°C, as measured by a built-in digital thermometer with an accuracy of 0.1°C. Mice were placed on the hot plate (25.4 cm × 25.4 cm), which was surrounded by a clear acrylic cage (19 cm tall, open top), and the Start/Stop button on the timer was activated. The latency to the hindlimb response was measured to the nearest 0.1 s by manually stopping the timer when the response was observed. The mouse was immediately removed from the hot plate and returned to its home cage. Animals were tested one at a time and were not habituated to the apparatus prior to testing. Each animal was tested only once. The latency to response for each mouse was recorded and the average latency to hind paw response ±SEM. determined for each group. These values were compared statistically by Student’s *t*-test (two groups) or by one-way ANOVA (three or more groups) and considered significantly different if the *p*-value was less than 0.05. An animal receiving the requisite volume of vehicle were alternated with each mouse given the test drug (*n* = 8 per group).

### COMPUTATIONAL MODELING AND ANALYSIS

Homology model of human NTS1 receptor was built using knowledge-based method. The crystal structure of NTR1 from *Rattus norvegicus* [PDB entry 4GRV (White et al., 2012)] was selected as template (with 89% sequence identity) and homology-modeling exercise was performed using the Prime module (version 3.1) of Schrödinger Suite (Schrödinger, 2012). The built model was refined using molecular mechanics based energy minimization and molecular dynamics simulation was performed for further structural analysis and characterization. Due to the high homology of the NTR1 and NTS1 at the binding site (94% sequence identity) and the constrained peptide-binding channel, coordinates of the contulakin-G peptide (SNATKKPYIL) were initialized using the coordinates from the NT crystal structure [PDB entry 4BUO (Egloff et al., 2014)]. Only the C-terminal residues (RRPYIL) of the NT crystal structure were used for initialization with the Arg residues were substituted for Lys and the rest of the peptide (N-terminal) was constructed using xLEaP module of Amber package (Case et al., 2012). The generated model peptide was minimized in vacuum without altering the rest of the system. To understand the effect of glycosylation (β-D-Gal(1→3)-αD-GalNAc-(1→)) of the Contulakin-G on binding to NT receptor, two systems were created with (a) glycosylated and (b) de-glycosylated Contulakin-G. The disaccharide moiety was attached to the Thr10 position of Contulakin-G and geometry optimized using the online carbohydrate builder web-server for Amber-Glycam force field (). Both systems were solvated using a pre-equilibrated box of TIP3P water model (Jorgensen et al., 1983) maintaining a minimum distance of 15 Å between any protein atom and the edge of the box. The final orthorhombic box contained a total of 54,596, or 54,617 atoms for the de-glycosylated or glycosylated system, respectively. To neutralize the system, ten Cl- ions were added to the simulation box, with parameters compatible with the AMBER force field and the TIP3P water model (Joung and Cheatham, 2008).

Molecular dynamics (MD) simulation was performed using the GPU version of the AMBER12 simulation package (Case and Kollman, 2012; Gotz et al., 2012). During the initial stage, the solvent atoms around the protein were relaxed by minimizing the system using 1000 steps of steepest descent algorithm. The protein heavy atoms were restrained during the minimization step with a harmonic potential of 100 kcal/mols/Å^2^. Following minimization, both systems were heated in the NVT ensemble from 150 to 300 K in 50 ps using time-steps of 1 fs. The protein backbone atoms were restrained using 1 kcal/mol/Å^2^ force constant during the heating step and velocities were randomly initialized from a Maxwell–Boltzmann distribution at 150 K. In all cases, bonds involving hydrogen atoms were restrained using the SHAKE algorithm with a geometric tolerance of 0.0001 Å (Miyamoto and Kollman, 1992). Periodic boundary conditions were imposed and the Particle Mesh Ewald (Darden et al., 1993) summation was used to approximate the electrostatic interactions (real-space non-bonded interaction truncated at 8.0 Å). During the initial 5 ns equilibration phase, simulations were performed in the NpT ensemble with a 2 fs time step and using a reference temperature of 300 K controlled through a Langevin thermostat (2.0 ps^-1^ collision frequency; Pastor et al., 1988) The system pressure was maintained around 1 atm using an isotropic weak-coupling algorithm (Berensen et al., 1984; 5 ps relaxation time). Both deglycosylated and glycosylated systems were simulated for 30 ns and snapshots were saved every 10 ps for analysis. Conformations from last 20 ns of the simulation were used for estimating the binding free energy using MM/PBSA (Molecular mechanics/Poisson–Boltmann Surface area) approach using the single trajectory method (Massova and Kollman, 2000; Miller et al., 2012).

## RESULTS

### AGONIST PROPERTIES OF CONTULAKIN-G AND NT

**FIGURE 2:**
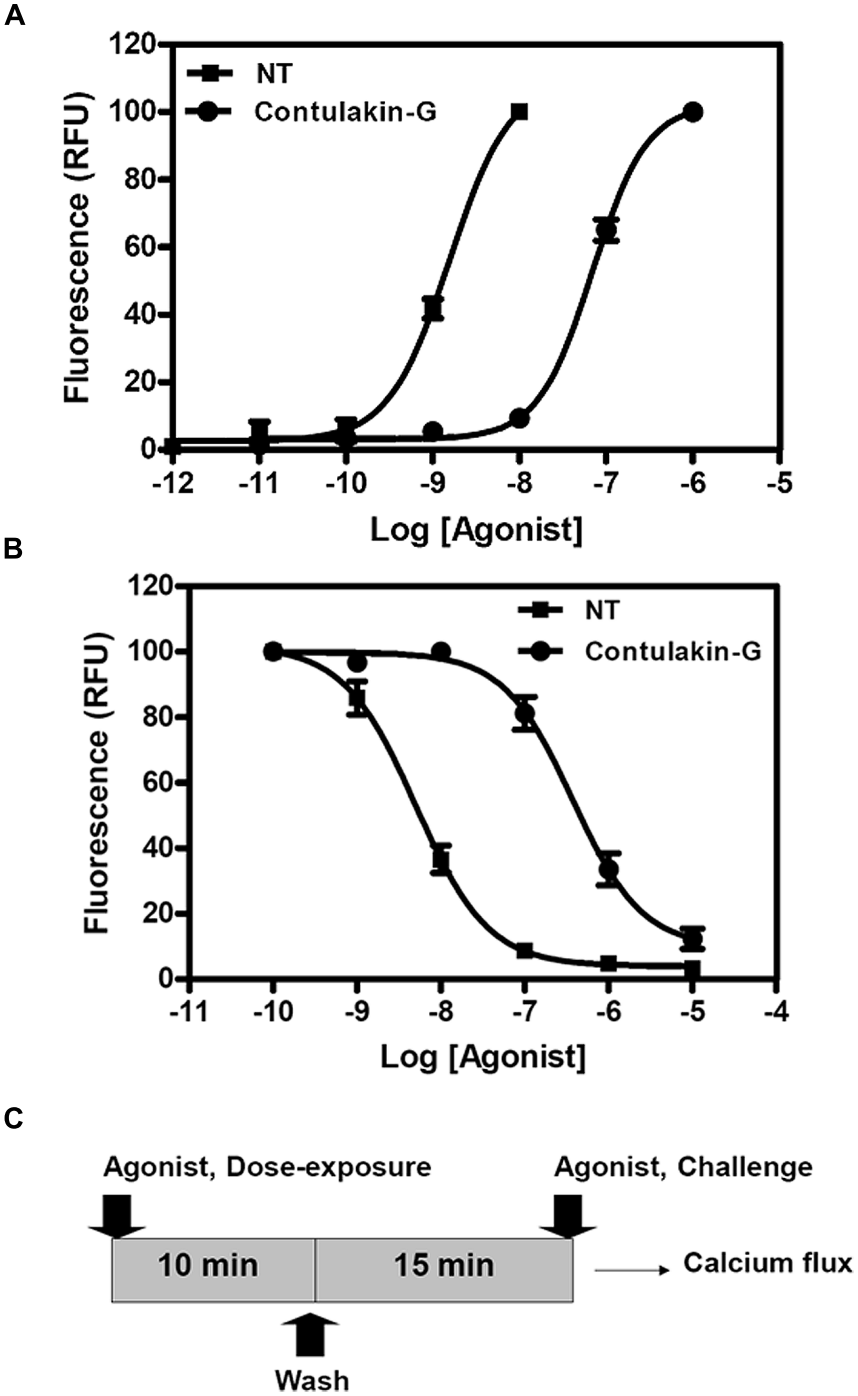
**Comparing agonist properties of NT and Contulakin-G. (A)** Concentration-dependent changes of the intracellular calcium in HT-29 cells that express high levels of NTS1. The potency values EC_50_ were 32.4 ± 14.5 nM and 0.8 ± 1.0 nM for Contulakin-G and NT, respectively. RFU stands for Relative Fluorescence Units, expressed as a percentage of the 1 μM peak response. The baseline was similar for both NT and Contulakin-G.** (B)** Representative dose-response curves in the desensitization assay. Contulakin-G (DC_50_ = 444.0 ± 40.8 nM) was 120-times less potent than NT (DC_50_ = 3.7 ± 1.9 nM) in desensitizing neurotensin receptors. The results were expressed as a percentage of the 1 μM peak response in the control cells (not previously exposed to the agonist). **(C)** Schematic representation of the desensitization assay.

To compare agonist activities of Contulakin-G and NT, we measured intracellular calcium mobilization in human colorectal cancer cell line HT-29, which is known to express high levels of NTS1. After incubation of the cells with Fluo-4-NW, the fluorescence emission due to intracellular calcium mobilization elicited by exposure to the peptide agonists was determined. The dose-response experiments for Contulakin-G and NT are shown in **Figure 2A**, yielding EC_50_ = 32.4 ± 14.5 nM and EC_50_ = 0.8 ± 1.0 nM, respectively. These results confirmed previous findings that Contulakin-G was a weaker agonist for the neurotensin receptors, as compared to that of NT (Craig et al., 1999).

**FIGURE 3:**
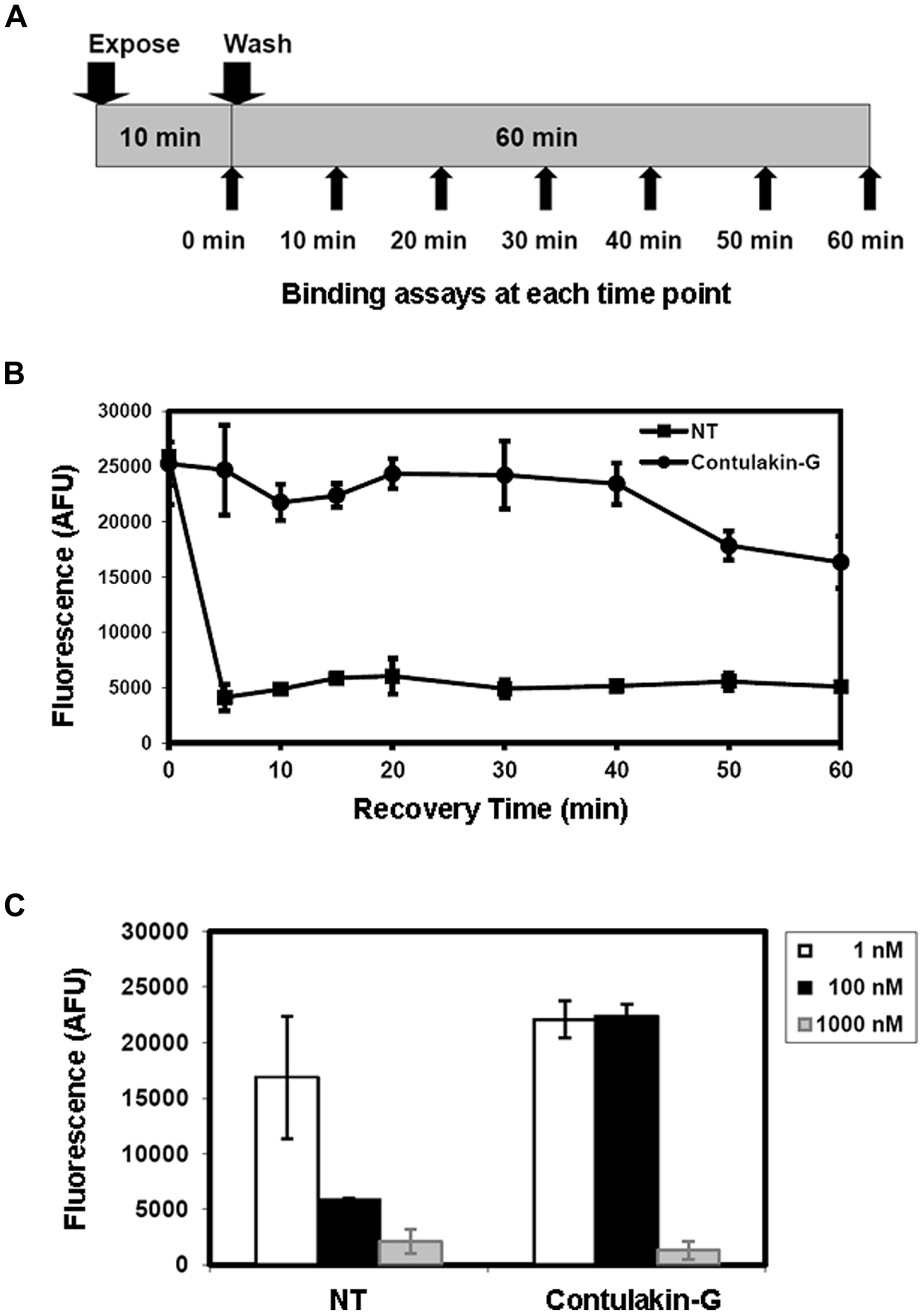
**Effects of Contulakin-G and NT on cell-surface neurotensin receptors. (A)** Schematic representation of the experiment measuring surface-bound neurotensin receptors upon exposure to the agonist. HT-29 cells were exposed to 100 nM of agonist for 10 min. Then, at the indicated time, cells were washed, and the binding assay was performed using Eu-NT as a ligand. **(B)** The time-dependence of the surface-bound neurotensin receptors upon exposure to the agonist. The higher fluorescence shows more NTR is available on the cell surface. AFU stands for Arbitrary Fluorescence Unit determined by the fluorescence microplate reader. **(C)** The NTR density on cell surface upon the agonists exposure at various agonist concentrations.

Next, we tested the hypothesis that weak agonist properties of Contulakin-G would result in inducing less desensitization of the target receptors, as compared to that of NT. To compare desensitization properties of Contulakin-G and NT, we developed an assay in which the cells were exposed to a 10 min pre-treatment with varying concentrations of the agonist, followed by the wash step. Then, the washed cells were treated with a pulse of agonist, followed by measuring the intracellular calcium mobilization. The concentration-response curves and the scheme summarizing the desensitization experiments are shown in **Figures 2B,C**, respectively. Contulakin-G (DC_50_ = 444.0 ± 40.8 nM) was 120-times less potent than NT (DC_50_ = 3.7 ± 1.9 nM) in the desensitization assay (**Table 1**). To further investigate differences in the desensitization of neurotensin receptors upon exposure to Contulakin-G or NT, we determined the availability of the cell surface neurotensin receptors. The experimental design (**Figure 3A**) was similar to that of the desensitization assay (10 min exposure to the agonist, followed by agonist washout), however, we employed the receptor-binding assay at various time points post-exposure, instead of measuring the intracellular calcium mobilization. As shown in **Figure 3B**, significantly higher levels of the cell-surface neurotensin receptors were observed when the cells were exposed to 100 nM Contulakin-G, as compared to the same concentration of NT. At either higher or lower concentrations of both agonists, the differences in changes of the surface-bound receptors were smaller (**Figure 3C**). Our findings suggested that the weaker agonist properties of Contulakin-G resulted in less ability to desensitize the receptors by preserving their cell-surface density.

**Table 1:** The structure-desensitization relationships for NT and Contulakin-G in activating the neurotensin receptors.

Name	EC_50_ [nM]	DC_50_ [nM]	DC_50_/EC_50_	Sequence
**Neurotensin**
NT	0.8 ± 1.0	3.7 ± 1.9	4.6	ZLYENKPRRPYIL
[K8, K9] NT	20.2 ± 5.2	67.0 ± 18.1	3.3	ZLYENKPKKPYIL
[A4] NT	0.6 ± 0.1	11.7 ± 1.4	20	ZLYANKPRRPYIL
[K4] NT	1.4 ± 0.1	30.5 ± 6.2	22	ZLYKNKPRRPYIL
[E6] NT	4.7 ± 1.4	57.5 ± 5.9	12	ZLYENEPRRPYIL
**Contulakin-G**
Contulakin-G	32.4 ± 14.5	444 ± 41	14	ZSEEGGSNAT_ (g)_KKPYIL
[T10] Contulakin-G	1.1 ± 0.7	144 ± 17	131	ZSEEGGSNATKKPYIL
[des GGS] Contulakin-G	15.0 ± 4.1	329 ± 49	22	ZSEENATKKPYIL
[Ahp5] Contulakin-G	4.2 ± 3.0	706 ± 160	168	ZSEE(Ahp)SNATKKPYIL
palmitoyl-Contulakin-G	46.0 ± 8.3	4.2 ± 0.8	0.1	ZSEEGGSNKK_(p)_KKPYIL
memantine-Contulakin-G	43.2 ± 13.1	1506 ± 517	35	ZSEEGGSNKE_(m)_KKPYIL
JMV-449	1.1 ± 0.2	11.8 ± 2.0	11	K(ψCH_2_NH)KPYIL

*g, glycosyl; p, paltimoyl; m, memantine; Ahp, aminoheptanoic acid; ψCH_2_NH, pseudo peptide bond.*

### STRUCTURAL DETERMINANTS OF DESENSITIZATION PROPERTIES OF CONTULAKIN-G AND NT

Previous work showed that the removal of the glycosylated residue from Contulakin-G, [Thr10]Contulakin-G, resulted in 16 to 25-fold increase in the binding affinity to NTS1, while concurrently improving the agonist potency of the deglycosylated analog (Craig et al., 1999). As summarized in **Figure 1**, the presence of the glycoamino acid and the extended N-terminus are the two most apparent structural differences between Contulakin-G and NT. In addition, an alignment of NT sequences pointed to the conserved Lys6 residue, which was absent in Contulakin-G. Our structure-desensitization relationship study (summarized in **Table 1**) employed several NT analogs with replacements of the positively charged residues, as well as several Contulakin-G analogs varying in the length of the N-terminus, or having various non-natural amino acid residues at position 10. The SAR results for NT suggested that removing the negatively charged residue in position 4, did not affect its agonist potency, but decreased its desensitization potency by approximately fivefold. The Glu-replacement of the conserved Lys6 decreased the agonist potency (sixfold) whereas its desensitization properties (defined as a ratio DC_50_/EC_50_) increased only twofold, as compared to that for NT. The double replacement of the conserved Arg residues affected the agonist potency, but not desensitization properties of the NT analog. These data suggested a role of electrostatics in determining the receptor-agonist interactions.

Our SAR studies for Contulakin-G confirmed the importance of the glycoamino acid in determining interactions with neurotensin receptors (**Table 1**). The deglycosylated analog of Contulakin-G, [T10]Contulakin-G, exhibited comparable agonist potency to NT, whereas had strikingly different desensitization properties. This uncoupling of the agonist and the desensitization properties was further emphasized in the analog in which the Gal-GalNAc-Thr10 was replaced with a lipoamino acid. The Contulakin-G analog containing palmitoyl-Lys10 residue, palmitoyl-Contulakin-G, had comparable agonist potency to that of the glycosylated analog, however, its desensitization potency was different by two orders of magnitude. Shortening the length of Contulakin-G to that of NT (13-AA) by a central removal of three neutral residues, Gly5-Gly6-Ser7, in the analog [desGGS]Contulakin-G, did not significantly change its agonist properties. This was further confirmed by replacing the Gly5-Gly6-Ser7 fragment with a backbone spacer, amino-heptanoic acid [Ahp5]Contulakin-G.

### STRUCTURAL ANALYSIS OF BINDING OF CONTULAKIN G PEPTIDE AND NTS1 RECEPTOR USING COMPUTATIONAL MODELING

To better understand a role of glycosylation on decreasing the potency of Contulakin-G, MD simulations were performed on the glycosylated and de-glycosylated Contulakin-G interacting with human NTS1 receptor. Due to the unavailability of the human neurotensin crystal structure, the receptor was built using homology-modeling exercise [Prime module, Schrodinger Suite (Schrödinger, 2012)]. Macromolecular docking of peptides and protein are often limited due to inherent challenges in enumerating the degrees of freedom for the ligand and in addition to presence of non-standard post-translational modification. Introduction of post-translational modification of substrate might also induce conformational changes in protein receptor that are best represented using explicit solvent MD simulation (Moreira et al., 2010).

**FIGURE 4:**
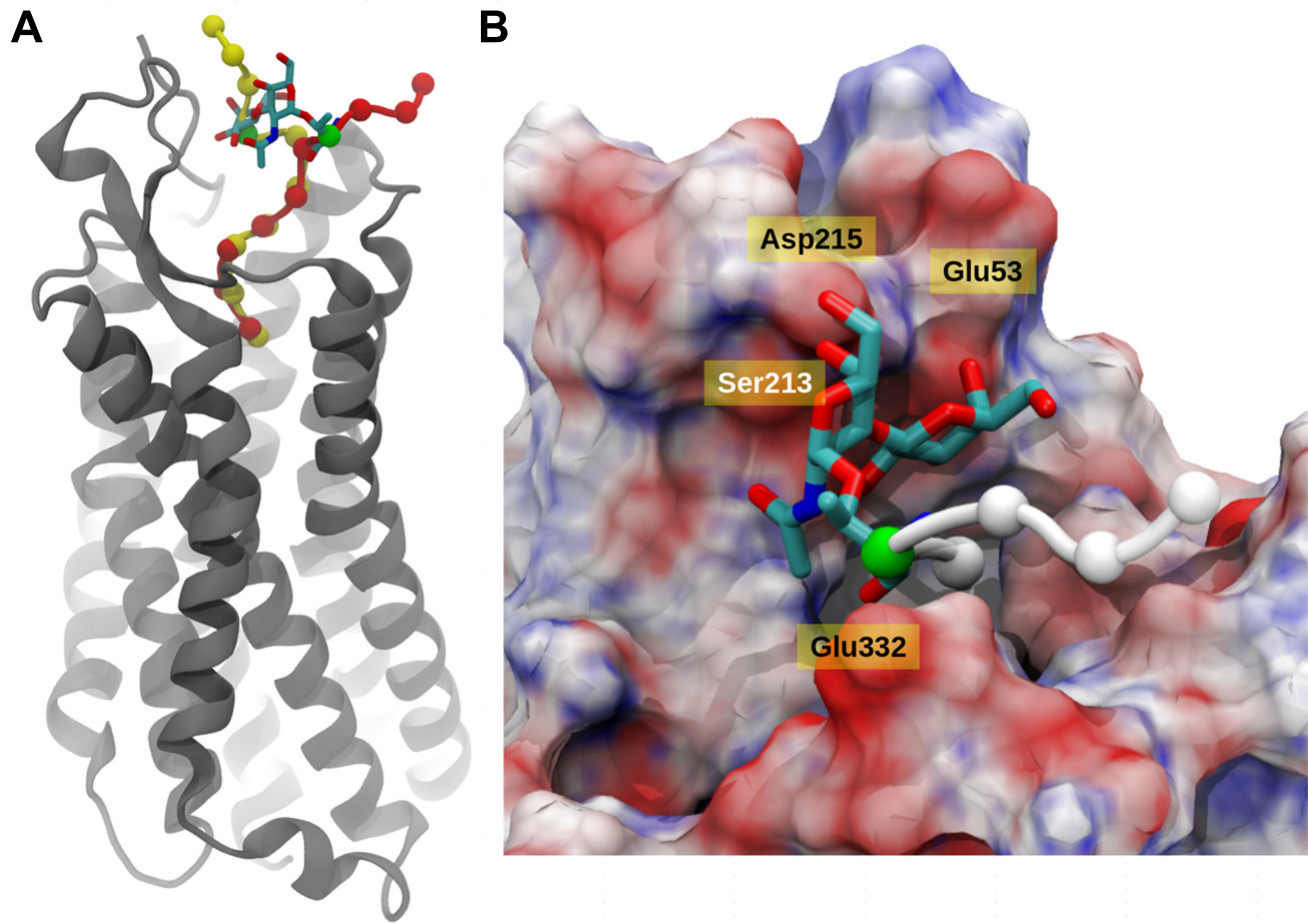
**Neurotensin receptor interacting with Contulakin-G. (A)** A snapshot from MD simulation of Contulakin-G interacting with NTR1 receptor, both glycosylated (red) and non-glycosylated peptides (yellow) are shown using van der Waals model with C^α^ atoms only, while NTR1 receptor (gray) is depicted using cartoon representation. In both cases, the glycosylating residue (Thr10) residue is colored green and the glycosyl moiety is shown using licorice model. **(B)** Top view of the binding site, with NT receptor colored using electrostatic potential (positively charged as blue, negatively charged as red using APBS software). The disaccharide moiety of Contulakin-G atoms are shown using licorice model while the peptide is shown using C^α^ atoms only (white). The rest of the peptide buried inside is not visible due to surface masking.

The integrity of the system was verified using the root mean square deviation (Figure S2) from the reference structure and it was clear that deglycosylated system showed lesser deviations compared to glycosylated system. In principal, the main deviations were observed at the N-terminal region of the peptide and its surrounding residues. Based on the 30 ns of explicit solvent MD simulation, it was evident that in both cases, the C-terminal six residues (KKPYIL) interacted with the NTS1 receptor identically (see **Figure 4A**). The C-terminal of the deglycosylated peptide made favorable interactions via salt bridge formation between carboxyl moiety of the peptide and the two-arginine residues (Arg94 and Arg241) of the receptor. These arginine residues are internally stabilized by cation-pi interactions provided by neighboring Tyr145 residue. This formation of salt bridge between the peptide and receptor defined the anchoring point for the C-terminal peptide and positioned Lys12 of the deglycosylated peptide to form hydrogen bond with Glu332. In addition to this; the N-terminal residues of deglycosylated peptide (SNAT) formed various hydrogen bond with the extracellular loop residues (Ser213–Asp215) and Ala48–Glu58 during the course of the simulation. Although, both peptides exhibited very similar interactions at the C-terminal region, glycosylation of Thr10 residue altered the conformation of the rest of the peptide significantly as compared to deglycosylated peptide. The key interaction between Lys12-Glu332 is lost due to restructuring of the N-terminal region and the backbone-hydrogen bond network between the extracellular loop and the N-terminal region is disrupted due to the presence of bulky glycosyl modification. Also, the glycosyl moiety is positioned close to the extracellular loop (Ser213–Asp215) is presented with various unfavorable interactions comparatively. Based on the electrostatic surface mapping using Poisson–Boltzmann software, the glycosylated moiety was seen anchored close to the negatively charged region (Asp215, Glu53, and backbone carbonyl group of the extracellular loop Ser213–Asp215) of the NTS1, and in turn restrained due to electrostatic repulsion (**Figure 4B**).

In the absence of direct comparison between experiment and computational approach, qualitative trend was assessed using estimation of binding free energy using simulation. To further gain insight into molecular interactions of Contulakin-G, free energy of binding (ΔG) was estimated using MM-PBSA approach. Using conformations from MD simulation, ΔG for both systems were calculated. The de-glycosylated and glycosylated analogs of Contulakin-G interacted with NTS1 receptor with ΔG of –90.00 and –57.68 kcal/mol, respectively (**Table 2**). Computational analysis predicted that the de-glycosylated peptide interacted with NTS1 with a much higher affinity compared to the glycosylated Contulakin-G. Analysis of the energetic components contributing toward the free energy revealed that the van der Waals and non-polar solvation energies were similar in both systems. However, the electrostatic and polar solvation energies differed significantly (**Table 2**), therefore pointing that the presence of the disaccharide moiety may confer electrostatic repulsion with the NTS1, in-line with the decreased agonist potency observed in the experiments with the glycosylated Contulakin-G.

**Table 2:** Binding free energies from MM-PBSA calculation.

	Energies (kcal/mol)	
	De-glycosylated	Glycosylated
ΔE (vdw)	–85.98	–87.02
ΔE (Elec)	–441.07	–360.58
ΔE (Polar Solv)	446.32	400.34
ΔE (Non-polar Solv)	–9.28	–10.41
ΔG (Total)	–90.00	–57.68

*Conformations from MD ensemble were used for free energy calculation.*

### CONVERTING CONTULAKIN-G INTO A PERIPHERALLY ACTIVE ANALGESIC WITH NON-DESENSITIZING PROPERTIES

Contulakin-G is a very potent analgesic in the formalin assay following direct administration into the CNS (Allen et al., 2007). Previous studies showed the analgesic efficacy of Contulakin-G in the tail flick latency test (Wagstaff et al., 2000), similarly to other NT analogs (Boules et al., 2006). Contulakin-G and NT are not systemically active analgesics, likely due to poor penetration across the BBB and/or high susceptibility to proteolytic degradation. Our studies to compare proteolytic degradation of Contulakin-G and NT in brain homogenates or serum (Supplemental Material) confirmed previous findings that the glycoamino acid protected Contulakin-G from degradation (Wagstaff et al., 2000). Due to its polar character, the glycosylation could also contribute to a limited penetration of Contulakin-G across the BBB and a lack of systemic activity as an analgesic, given that the antinociceptive activity of NT analogs is mediated by neurotensin receptors located in the CNS (Dobner, 2006; Boules et al., 2013; Kleczkowska and Lipkowski, 2013). We hypothesized that replacing a glycoamino acid residue in Contulakin-G with a non-natural and more hydrophobic residue would retain its weaker-agonist and desensitization properties while improving its systemic activity.

**FIGURE 5:**
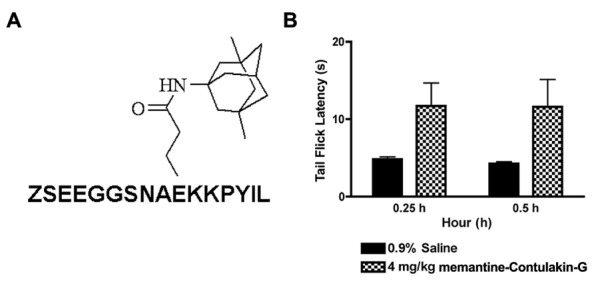
**The structure **(A)** and the analgesic activity of memantine-Contulakin-G analog following an intraperitoneal administration of 4 mg/kg bolus dose **(B**).** Tail flick latency was measured at 15 min and 30 min time points following administration of the analog.

To test our hypothesis, we designed and synthesized a novel Contulakin-G analog, memantine-Contulakin-G containing memantine coupled to Glu10 (**Figure 5A**). Memantine is a non-competitive *N*-methyl-D-aspartate (NMDA) receptor partial antagonist, and it was selected due to its hydrophobic nature and relatively small size, favoring the BBB penetration. The chemical synthesis of this analog is summarized in Supplemental Information and described in Methods. The agonist potency of memantine-Contulakin-G was comparable with that of Contulakin-G, however, its desensitization properties were approximately threefold weaker, yielding an analog with a 35-fold separation of the agonist and desensitization potencies (as defined by DC_50_/EC_50_; **Table 1**). Both peptides were tested in analgesic tests in mice, the hot plate and the tail flick assays at a single dose 4 mg/kg. As shown in **Figure 5B**, and summarized in **Table 3**, memantine-Contulakin-G was active in the tail flick assay, however, its analgesic activity in the hot plate assay was unclear due to the activity of memantine-alone, as a control. Memantine-Contulakin-G, at doses 8–20 mg/kg, i.p., was also active in suppressing seizures in the 6 Hz (32 mA) mouse model of epilepsy while showing no rotorod toxicity (unpublished data), further confirming its improved CNS bioavailability.

**Table 3:** Comparing analgesic activities of Contulakin-G analogs containing either a disaccharide or memantine in position 10.

	Tail flick latency	Hot plate
Contulakin-G	4.06 ± 0.37	4.94 ± 0.79
Memantine-Contulakin	11.59 ± 3.54**	8.09 ± 1.06*
Memantine	4.836 ± 0.264	7.9 ± 0.49*
Saline	4.68 ± 0.42	5.412 ± 1.9

The analogs were administered i.p., at a dose 4 mg/kg.

*p < 0.05, **p < 0.01.

## DISCUSSION

Contulakin-G is a marine natural product which targets neurotensin receptors and exhibits potent analgesic activities. This peptide reached clinical testing for the treatment of neuropathic pain in spinal cord injury patients, thus becoming one of several conotoxin-based therapeutic agents with the Investigational New Drug (IND) status. Several key characteristics of Contulakin-G are: (1) it is a very potent analgesic compound following intrathecal delivery in animal models of pain (Wagstaff et al., 2000; Allen et al., 2007; Han et al., 2008), (2) its analgesic activity is mediated by neurotensin receptor (Business Wire, 27 September 2005), (3) it is a weaker than NT agonist with the decreased ability to desensitize neurotensin receptors (Craig et al., 1999, and this work). Unlike all previously characterized analgesic conotoxins, Contulakin-G has no disulfide bridges, making this linear peptide susceptible to rapid proteolytic degradation. Previous studies (Wagstaff et al., 2000) and our current work showed that Contulakin-G maintains high resistance to proteolysis in both serum and brain homogenate media (Figure S3). Pharmacokinetic studies of Contulakin-G confirmed significant concentrations of the peptide several hours after bolus injections (Kern et al., 2007). One conclusion from this study is that Contulakin-G is a potent *in vivo* analgesic by being a metabolically stable and long-lasting agonist which induces less desensitization of target neurotensin receptors.

One translational aspect of developing Contulakin-G as NT-based analgesics is its lack of efficient penetration across the BBB (Wagstaff et al., 2000; Allen et al., 2007). This fact raises a more general question about preclinical and clinical development efforts of NT-based agonists for CNS indications. There are several BBB-penetrant NT analogs which exhibit analgesic activities following systemic administration, for example NT69L (Boules et al., 2006), ANG2002 (Demeule et al., 2014), or polyamine-NT (Zhang et al., 2009). BBB-penetrant and NTS1-selective agonist, PD149163 (Wustrow et al., 1995), was developed by Pfizer and is active in drug self-administration test (Hanson et al., 2013) and in cognitive performance test (Keiser et al., 2014). Another example of BBB-penetrant NT analog is the anticonvulsant NT-BBB-1, which was active in a pharmacoresistant model of epilepsy (Green et al., 2010). Thus, from a translational perspective, systemically active NT analogs which exert CNS effects seem more attractive as drug leads, as compared to that of Contulakin-G, which requires intrathecal delivery. In our current study, we provided a proof-of-concept example of designing Contulakin-G analog (memantine-Contulakin-G) which maintains weak agonist and desensitization properties while producing analgesia in the tail flick assay following systemic administration. Our structure-desensitization relationship data (**Table 1**) open new strategies for designing NT-analogs which can penetrate the BBB and exhibit diverse abilities to desensitize neurotensin receptors. However, systemic administration of the BBB-permeable NT analogs to target the CNS has to overcome the safety challenges, given a role of NT in promoting cancer progression (Dupouy et al., 2009; Alifano et al., 2010; Wu et al., 2013; Younes et al., 2014; Zhang et al., 2014). Possible cancer-enhancing activities of the systemically active NT analogs should be carefully evaluated when considering IND-enabling studies for both acute and chronic indications. One possible strategy to mitigate the safety-related undesirable activities is to generate and examine highly subtype-selective NT agonists for NTS1, NTS2, and NTS3, or for specific receptor heterodimers (our preliminary efforts to generate peptoid-based analogs of Contulakin-G with more selectivity toward NTS2 suggested a loss of the agonist activities of the hybrid analogs at concentrations up to 3 μM, unpublished data).

Our SAR and computational modeling data suggested that differences between Contulakin-G and NT as agonists can be accounted for, in large part, by the presence of glycosylation. Glycosylation of neuropeptides is rather uncommon post-translational modification, as compared to larger polypeptides and proteins. Contulakin-G from a marine cone snail, bradykinin from wasps and somatostatin from a catfish are three known examples of naturally glycosylated neuropeptides targeting GPCRs (Yoshida et al., 1976; Rocchi et al., 1987; Gobbo et al., 1992; Piek et al., 1993; Craig et al., 1999; Chen et al., 2000). All three peptides share similar glycosylation pattern: O-glycosylated threonine with a galactosamine-galactose moiety, β-D-Gal-(1→3)-α-D-GalNAc-(1→) Thr, also found in Thomsen-Freidenreich antigens (TF-antigens) expressed on a surface of cancer cells (van den Akker et al., 1996; Gambert and Thiem, 1997; Glinsky et al., 2001; Siebert et al., 2002; Kunz, 2003). Our modeling study predicted that the presence of the glycoamino acid significantly decreased the free binding energy to neurotensin receptor, also affecting electrostatic interactions between the peptide and receptors. It is tempting to hypothesize here that despite weakening interactions with the target receptors [to make a prey fish more sluggish as a part of “nirvana” cabal (Olivera, 1997; Olivera et al., 1999)], the glycosylation of Contulakin-G offers an evolutionary advantage for *C. geographus*, due to protecting this secreted peptide from metabolic degradation.

This work opens new opportunities in engineering NT-based analogs with varying abilities to modulate agonist-induced desensitization of neurotensin receptors, a well-characterized phenomenon that has been shared by GPCRs including neurotensin receptors (Vincent et al., 1999; Pelaprat, 2006). In our study, we employed HT29 cells endogenously expressing NTS1 receptors which activation results in the calcium mobilization and stimulating inositol triphosphate pathways (Amar et al., 1986; Turner et al., 1990). The trafficking of NTS1-NT complex varies according to a cell type, as well as time of exposure to the agonists (Vandenbulcke et al., 2000; Souaze, 2001; Mazella and Vincent, 2006). Short-term (minutes) exposure to NT, such as that applied in our experiments, caused the receptor activation, uncoupling of NTS1 from G proteins, binding to β-arrestin, then endocytosis (Souaze and Forgez, 2006). Noteworthy, a chronic exposure of NTS1 to high concentrations of an agonist had different effects; 6 h exposure to 100 nM of the agonist JMV449 significantly upregulated levels of NTS1 mRNA. After 48 h, the cells maintained a high level of ^125^I-NT-binding sites, only approximately twofold lower as compared to that of the unchallenged cells (Souaze et al., 1997; Souaze and Forgez, 2006). Our current work confirmed that short-term exposure to weaker agonists may result in less desensitization of GPCRs (Clark et al., 1999). Noteworthy, at the highest concentrations of Contulakin-G (∼30-fold EC_50_), neurotensin receptors were internalized (**Figure 3C**). Based on the computer modeling data we hypothesize that additional SAR study focused on Lys12 may identify analogs with even less desensitizing properties. Several analogs of Contulakin-G studied here already produced differences of several orders of magnitude with respect to uncoupling the agonist and desensitization potencies. Our SAR results, while limited, suggest that desensitization of neurotensin receptors can be significantly uncoupled from the agonist potencies, and that substitutions in charged amino acid residues in NT analogs are attractive sites to engineer agonists with diverse desensitization potencies. These opportunities are supported by other findings, where MAS receptor ligands were reported to induce less desensitization (Tirupula et al., 2014). Non-desensitizing properties for agonist-based drug leads have direct relevance to their pharmacological properties, as exemplified by β2-agonists (Duringer et al., 2009) or salvinorin A (Groer et al., 2007; Cunningham et al., 2011).

In conclusion, Contulakin-G is a marine glycopeptide with analgesic properties by being a metabolically stable and weaker than NT agonist of neurotensin receptors, resulting in prolonged half-life and circulation while inducing significantly less desensitization of the cell-surface receptors. The analog of Contulakin-G in which the glycosylated residue was replaced by memantine had comparable agonist potency and weaker desensitization properties as compared to that of Contulakin-G, also producing analgesic activity following systemic administration. Our structure-activity relationship and computer modeling studies suggested that the replacements of the charged and glycoamino acid residues in Contulakin-G may lead to the systemically active NT analogs with diverse potencies for activating and desensitizing neurotensin receptors.

## Conflict of Interest Statement

Grzegorz Bulaj and H. Steve White are scientific cofounders of NeuroAdjuvants Inc., Baldomero M. Olivera and J. Michael McIntosh are scientific founders of Cognetix Inc.

## SUPPLEMENTARY MATERIAL

The Supplementary Material for this article can be found online at:
