# Supplementary material for: A marine analgesic peptide, Contulakin-G, and neurotensin are distinct agonists for neurotensin receptors: uncovering structural determinants of desensitization properties
Source: Front Pharmacol. 2015 Feb 10;6:11. doi: 10.3389/fphar.2015.00011 (PMC4322620; doi:10.3389/fphar.2015.00011)
Supplement: Supplementary file 1 [file Presentation_1.PDF]

SUPPLEMENTAL INFORMATION

A Marine Analgesic Peptide, Contulakin-G, and Neurotensin are Distinct Agonists for Neurotensin Receptors: Uncovering Structural Determinants of Desensitization Properties

Synthesis of memantine-Contulakin-G. In the synthesis of CG-Memantine, Glu^{10}(OAll) was automatically conjugated on Wang resin in the solid phase peptide synthesis (SPPS) during the assembling of all the other amino acids; then Allyl protecting group of Glu^{10} was removed by $Pd^0(PPh_3)_4$ with scavenger cocktail HOAc/N-methylmorpholine (NMM) in CH_2Cl_2, the resin was washed with CH_2Cl_2, neutralized with DIPEA; excess Pd residue was capped with 0.02 M sodium diethyldithiocarbamate in DMF solution, and the resin was washed again with CH_2Cl_2; 3-fold Memantine Hydrochloride, PyBOP, HOBt and DIPEA (1:0.98:1:2) were added to the resin, shaken for overnight, and then the peptide was cleaved with reagent K from the resin to give CG-memantine.

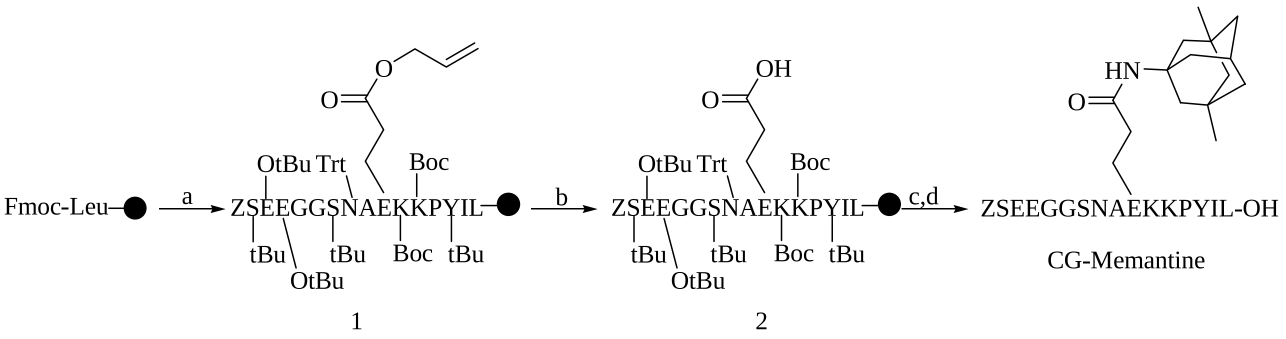

Figure S1. Synthesis strategy for Contulakin-G-Memantine. (a) Wang preload resin:(Fmoc-amino acid/Pybop/DIEA, 1/0.98/2), 1:5, 40 min; Fmoc was removed by 20% piperidine in NMP; (b) Resin:$Pd(PPh_3)_4$ in NMM/HAc/DCM (1/2/40), 1:5.5, 2 h; (c) coupling of memantine Resin/Memantine/PyBop/HOBt/DIEA, 1/1/1/1/2, 24 h; (d) Deprotection and removal from the resin by treating with reagent K (TFA/phenol/water/thioanisole/1,2-dithioethane, 82.5/5/5/5/2.5), 3 h. Final purity was determined by HPLC as >95%.

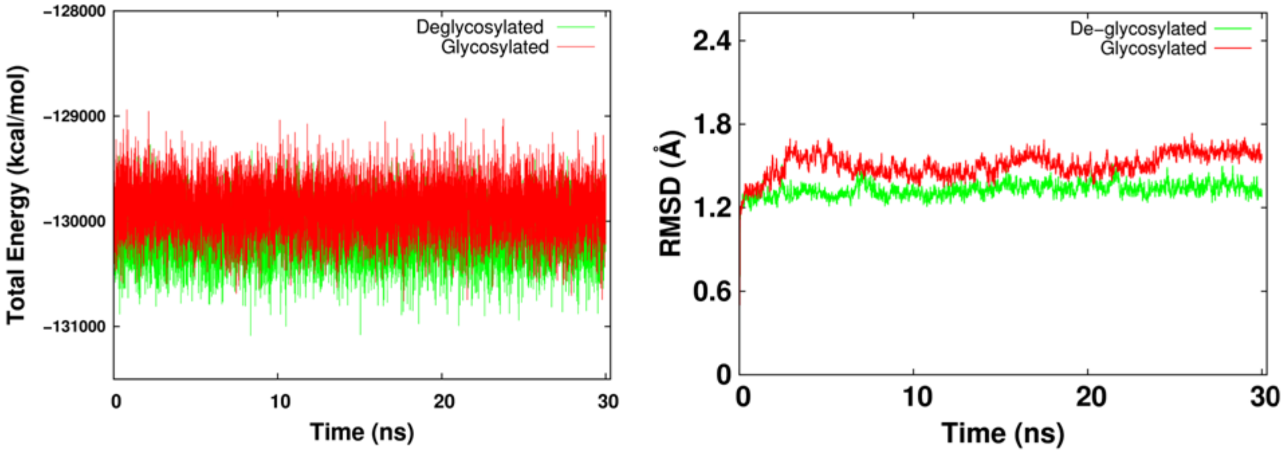

Figure S2: Molecular dynamics simulation of neurotensin receptor NTS1 in complex with contulakin-G peptide. RMSD and total energy data for glycosylated (red) and deglycosylated (green) system. RMSD was calculated using the backbone atoms after fitting the trajectory to the initial structure.

Metabolic stability of Contulakin-G and NT. We also compared in vitro metabolic stabilities of NT and Contulakin-G, hypothesizing that high in vivo potency of Contulakin-G as analgesic may result in part from a higher metabolic stability, in addition to favorable desensitization properties. Samples were assayed at a concentration of 0.25 µg µL^{-1} in triplicate. The samples were incubated at 37 °C in 20% rat serum. At the specific time point, 200 µL of the reaction mixture was removed, and added to 100 µL of TCA mix (15% trichloroacetic acid, 40% isopropanol) to precipitate serum proteins. After 15 min of incubation at -20 °C, the precipitate was spun down, and the supernatant was analyzed by reversed phase separations using YMC ODS-A S-5 120 Å column (AA12S052503WT) analytical HPLC columns. Aliquots of the samples were injected, and the amount of remaining intact analog was determined by integration of the RP-HPLC peaks recovered at 210 nm. For the brain stability assay, 10% mouse brain homogenate was used.

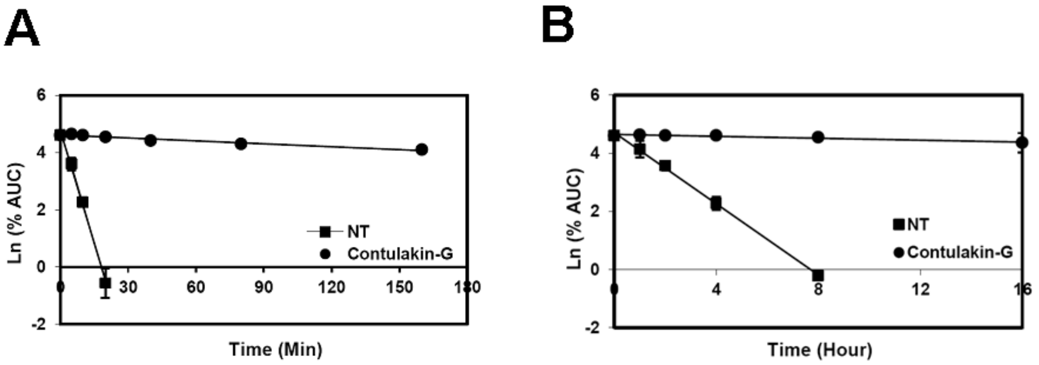

Figure S3. Metabolic stability assays in 10% mouse brain homogenate (A) and 20% rat serum (B). The metabolic stability assay in the brain homogenate yielded the half-lives for Contulakin-G and NT as >160 min and 3 min, respectively. The metabolic stability assay in the serum yielded the half-lives for Contulakin-G and NT >16 hrs and 1.3 hr, respectively.
